# Everolimus in Anaplastic Thyroid Cancer: A Case Series

**DOI:** 10.3389/fonc.2019.00106

**Published:** 2019-02-26

**Authors:** Ethan J. Harris, Glenn J. Hanna, Nicole Chau, Guilherme Rabinowits, Robert Haddad, Danielle N. Margalit, Jonathan Schoenfeld, Roy B. Tishler, Justine A. Barletta, Matthew Nehs, Pasi Janne, Julian Huang, Phillip Groden, Alec Kacew, Jochen Lorch

**Affiliations:** ^1^Dana-Farber Cancer Institute, Boston, MA, United States; ^2^Miami Cancer Institute, Miami, FL, United States; ^3^Brigham and Women's Hospital, Boston, MA, United States; ^4^Yale School of Medicine, New Haven, CT, United States; ^5^Icahn School of Medicine at Mount Sinai, New York, NY, United States

**Keywords:** anaplastic thyroid cancer (ATC), precision medicine, mTOR inhibition in head and neck cancer, exceptional responder, PI3K mTOR

## Abstract

**Background:** Anaplastic thyroid cancer (ATC) is a very aggressive disease and accounts for over 50% of thyroid-cancer related deaths. mTOR inhibition has shown anti-tumor activity in ATC. We report our experience treating patients with ATC with everolimus off-protocol.

**Methods:** Patients with confirmed ATC and treated with everolimus at DFCI were identified and reviewed retrospectively. NexGen sequencing was performed, and radiologic responses were correlated with mutational profile.

**Results:** Five patients were treated from 2013 to 2016. Three patients had a response, which included one patient who achieved a partial response for 27.9 months, and two patients who had stable disease for 3.7 and 5.9 months, respectively. Genomic analysis was available in two patients and revealed that the partial responder had mutations involving the PI3K/mTOR pathway.

**Conclusion:** Everolimus has anti-tumor activity in ATC, and responses may correlate with mutations involving the PI3K/mTOR pathway. Further studies are warranted.

## Introduction

Anaplastic thyroid cancer (ATC) constitutes only 1–2% of all thyroid cancer cases; however, it is one of the most lethal types of cancer and has a uniformly poor prognosis with a median survival of 3–5 months (1). It accounts for more than 50% of all thyroid cancer-related deaths due to its aggressive biology, which is characterized by early hematogenous metastasis. It may arise from differentiated thyroid cancer TC (2), occurs more frequently in men, and typically afflicts the elderly.

There is currently no standard treatment for metastatic ATC. Weekly taxane with or without platinum may be used, but these agents have an unknown impact on overall survival (3, 4). In patients with the activating BRAF^V600E^ mutation, the combination of the BRAF and MEK inhibitors dabrafenib and trametinib, has recently shown promising activity in ATC (5, 6). Aside from that group of patients, treatment success has been largely disappointing, highlighting the need for more effective treatment regimens.

Genomic analysis has found that ATC has a high mutational burden, which contrasts with the genomic landscape of differentiated thyroid cancer, such as papillary TC, and poorly differentiated thyroid cancer (PDTC) (7). In ATC, TERT, TP53, SWI/SNF subunits, histone methyltransferases, and the PI3K/mTOR/AKT pathway were frequently altered. Within the PI3K/AKT/mTOR pathway, mutations in PIK3CA, AKT, mTOR, PTEN, TSC1, TSC2, and NF1 were more common in ATC than PDTC, indicating the pathway plays a central role in disease progression (8).

Rapamycin and its analogs, such as everolimus, are allosteric inhibitors of the mTOR pathway and specifically inhibit activation of the mTOR complex 1. Everolimus is FDA approved to treat various cancers, including breast and renal cell carcinoma. We have previously treated seven patients with ATC in an exploratory cohort within a phase 2 study using everolimus and had two responses (9). Another phase 2 study using everolimus in patients with thyroid cancer included 6 patients with anaplastic histology, and one partial response was observed (10). Here, we report our experience with everolimus given off-protocol in 5 patients with ATC.

## Materials and Methods

### Chart Review

The DFCI's Institutional Review Board approved this retrospective case series and chart review for all patients with ATC who were treated with everolimus off-protocol. All patients provided written consent, and all patients from 2013 to 2016 were included in this analysis. Investigators assessed tumor response using radiologic imaging and confirmed them using the corresponding radiologist reports. Partial responses were calculated starting from the first scans demonstrating a response, and stable disease was calculated from the start of everolimus. Overall survival was calculated from the start of everolimus until death. All durations were calculated in months.

### Genomic Sequencing

Patients who provided written consent for tumor sequencing had fixed-formalin paraffin embedded slides prepared for total gDNA extraction. ATC pathology was confirmed prior to extraction. The sample's gDNA concentration was quantified and, if sufficient, underwent library construction, subjected to qPCR, and analyzed based on the OncoPanel_v1 bait set (Oncopanel), a database of 275 cancer genes and 91 introns across 30 genes with known or potential importance in cancer, using an Illumina Hiseq 2500. Picard tools, which align and de-multiplex read pairs, and the GATK tool, which localizes realignment, were used (11, 12).

### Tissue Diagnosis

An expert in thyroid pathology (JB) reviewed all ATC cases in this study and confirmed the diagnoses.

## Results

Five patients who had ATC and were treated with everolimus off-protocol were identified. Four patients were male and one was female; the median age at diagnosis was 75 (range: 62–79). Three patients had distant metastatic disease at the time of diagnosis, and two had unresectable locoregional disease. One patient had an associated component of papillary thyroid cancer in her tumor specimen. Two patients received carboplatin and paclitaxel with radiotherapy prior to starting everolimus, and one patient, who had papillary thyroid cancer 12 years prior to her ATC diagnosis, was initially treated with radioactive iodine (RAI), and then a resection in the thyroid bed for a local recurrence.

**Figure 1:**
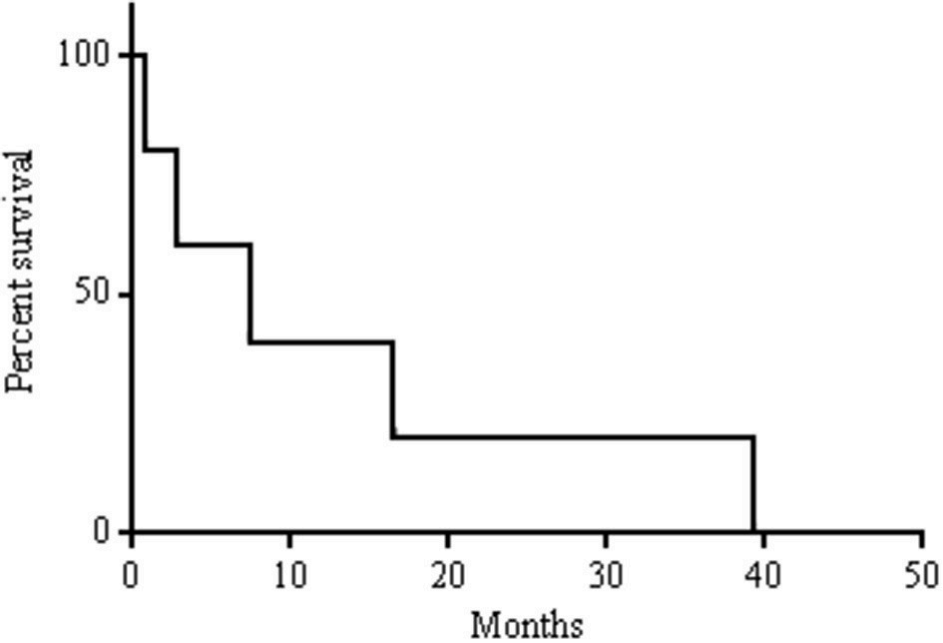
Kaplan-Meier survival curve.

**Figure 2:**
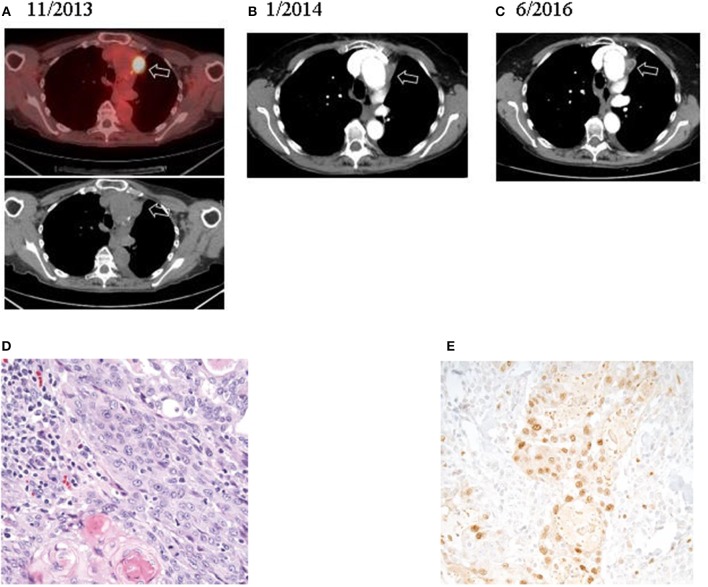
Radiologic and histologic imaging of the patient who achieved a partial response (#3).

All patients started treatment taking 10 mg of everolimus every day. Due to toxicities such as fatigue and pneumonitis, patients could receive 10 mg of everolimus every other day. Overall, patients took everolimus for a median of 5.3 months (range: 0.8–29.5). Overall, median survival in this cohort was 7.4 months (Figure 1). One patient had a partial response for 27.9 months. This patient's treatment course was complicated by mTOR-induced pneumonitis. Initially, the side effects from everolimus were managed with treatment breaks, but after 12.6 months of treatment, her dose was reduced to 10 mg every other day. This allowed her to continue with everolimus for another 16.9 months before her treatment was discontinued. CT scans revealed that her disease was well controlled at the time of discontinuation of the drug (Figures 2A–C). Two other patients had stable disease for 3.7–5.9 months, respectively. One patient had a mixed response, and another patient had disease progression.

**Figure 3:**
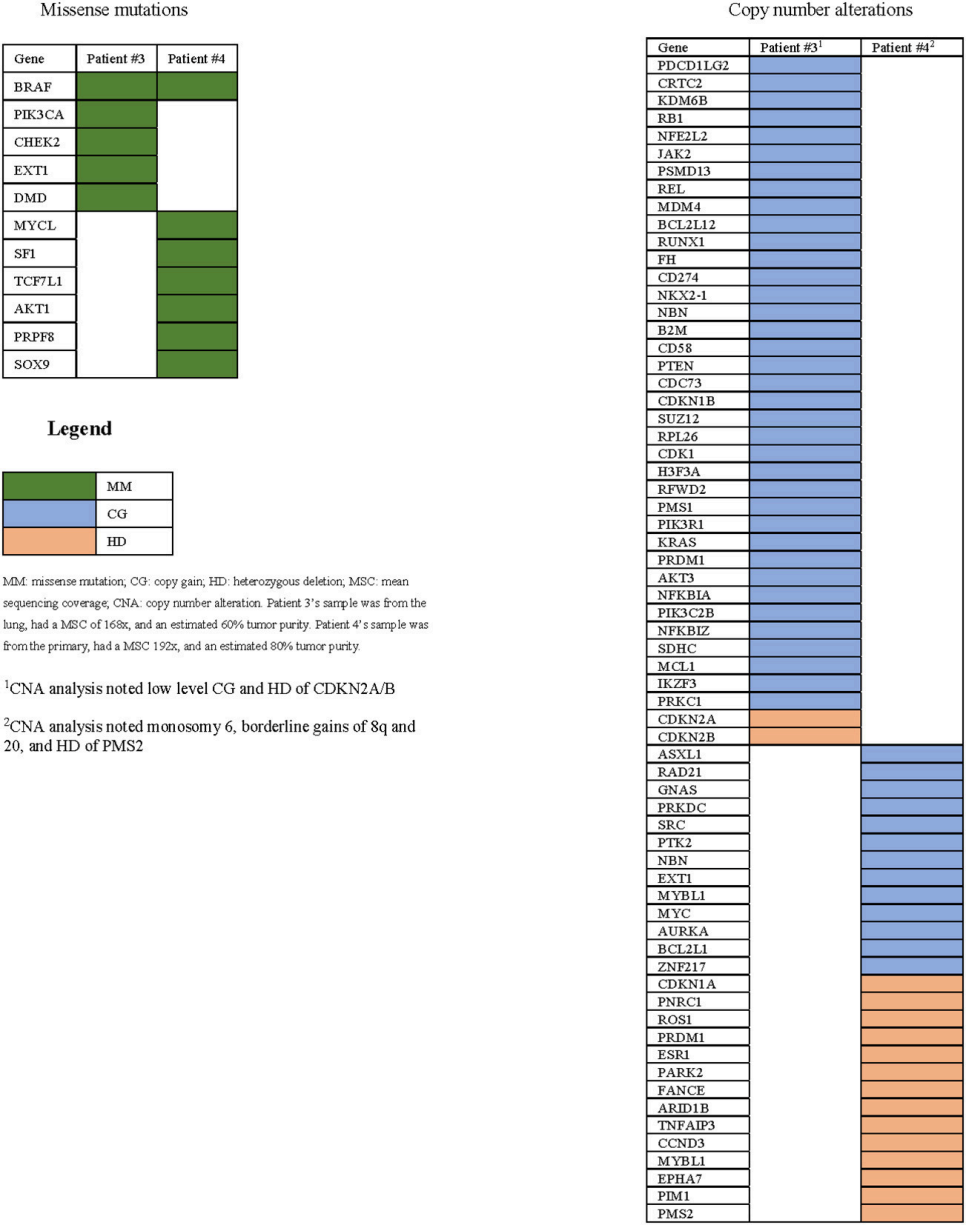
All genomic alterations of patients with sequencing data available.

Genetic analysis demonstrated the BRAF^V600E^ mutation and two known activating PI3K mutations (PIK3CA^E970K^ and PIK3CA^E542K^) in the partial responder. Additionally, the tissue's ATC diagnosis was confirmed (Figures 2D,E). Among the two patients who had stable disease, sequencing of the primary tumor revealed the BRAF^V600E^ mutation in one case; no sequencing could be performed in the other case due to lack of sufficient material (Table 1). Copy number alterations were also documented (Figure 3).

**Table 1:** Patient population and treatment results.

	1^*^	**2**	3^*^	4^*^	**5**
Histology	ATC	ATC	ATC with aPTC	ATC	ATC
Age at diagnosis (y)	75	62	79	78	63
Gender	Male	Male	Female	Male	Male
Stage	M	M	M	LR	LR
Prior treatment	None	Carboplatin and paclitaxel with radiation	RAI and thyroidectomy^c^	None	Carboplatin and paclitaxel with radiation
Mutations^a^	N/A	N/A	PIK3CA^E542K^	BRAF^V600E^	N/A
			PIK3CA^E970K^		
			BRAF^V600E^		
Duration prescribed everolimus (mo)	0.8	1.7^b^	29.5	5.3^d^	5.9
Best radiologic response, length (mo)	PD	MR	PR, 27.9	SD, 3.7	SD, 5.9
Overall survival (mo)	D, 0.8	D, 2.8	D, 39.2	D, 7.4	D, 16.5

* First-line of treatment

a *only actionable mutations included*,

b *EMR did not capture patient's final month alive, was not included in analysis*,

c *Treatment for PTC, prior to ATC diagnosis*,

d *Continued everolimus after radiologic PD, aPTC, associated component of papillary thyroid carcinoma; M, metastatic; LR, locoregional; RAI, radioactive iodine; PD, progressive disease; MR, mixed response; PR, partial response; SD, stable disease; D, deceased*.

## Discussion

ATC remains one of the deadliest forms of cancer with no generally accepted standard of care. In this retrospective analysis, we report 5 patients with ATC who underwent palliative treatment with everolimus. One patient had a partial response lasting for 27.9 months, and two patients had stable disease lasting 3.7–5.9 months, respectively. Overall, the median survival after diagnosis was 7.4 months in this cohort.

Chemotherapy is generally considered marginally effective, and documented responses to targeted therapies are rare. Tyrosine kinase inhibitors, which have significant activity in differentiated thyroid cancer (13–16), have been used with some success in ATC. In a report of 2 patients treated with Axitinib, one patient had a PR (17). In a phase II study with sorafenib, 4/10 patients with ATC had disease stability (40%) (18). Additionally, a multi-center trial found that 2/20 patients had a PR, lasting for 10–27 months, respectively, and 5/20 had SD (19). A single-arm phase II study using lenvatinib in thyroid cancers, including 17 patients with ATC, reported an objective response rate of 24% and median OS of 10.6 months (16). In a recent retrospective study of 5 patients with unresectable ATC treated with lenvatinib, Koyama et al. found that 3/5 had a PR, 5/5 had SD, and the cohort had an OS of 165 days (20). Pazopanib, a VEGF inhibitor, has weak activity in ATC; a mulitinistiutional study of 15 patients treated with pazopanib monotherapy did not have any confirmed RECIST responses (21).

Combination therapy with BRAF and MEK inhibitors has shown anti-tumor activity in ATC. A recently published clinical trial treating patients with BRAF^V600E^ positive ATC with dabrafenib (BRAF inhibitor) and trametinib (MEK inhibitor) reported a 69% ORR (11/16) with long-lasting responses (6). Another BRAF inhibitor, vemurafenib, has had some success (22, 23). In contrast, we have previously reported one patient with a BRAF^V600E^ and a PIK3CA^H1047R^ mutation who failed to respond to everolimus or dabrafenib and trametinib, but responded to the combined regimens (24).

In a prior phase II study, we evaluated everolimus as a single agent in 7 patients with metastatic ATC (9). Among these 7 patients, two responded: one achieved an exceptional response and another had disease stability for 31 months. Both patients carried mutations involving the PI3K/mTOR pathway; genetic analysis of the exceptional responder's tumor revealed a truncating TSC2 mutation, resulting in loss of negative regulation of mTOR signaling (25), and the other patient had a mutation in NF1, a regulator of TSC2 and mTOR, and in PIK3CA and mTOR (9).

Recent analyses have begun to elucidate the molecular drivers behind ATC tumorigenesis. TERT, TP53, and PIK3CA appear to be more frequently mutated than in other forms of thyroid cancer, including in poorly differentiated thyroid cancer (PDTC), which may present histologically and clinically similar to ATC. Sequencing 33 ATC and 84 PDTC tumor samples discovered that TERT and TP53 mutations have been reported in 73% of cases, but we did not find any in our samples. Additionally, PIK3CA mutations were present in 18% of ATC samples, but only in 2% of PDTCs, and PI3K/AKT pathway abnormalities were found in 39% of ATC samples vs. 11% of PDTC (8).

The partial responder's tumor was sequenced and revealed three somatic variants: PIK3CA^E542K^, a helical domain and hotspot mutation, PIK3CA^E970K^, a mutation in the PI3' kinase domain, and BRAF^V600E^, a common mutation in ATC (8, 26). Tumorigenesis in animal models indicate that activating PI3K mutations alone are not sufficient to trigger the development of ATC and therefore frequently occur in combination with other mutations, such as BRAF^V600E^. Cell culture data has demonstrated synergy in ATC when both genes were mutated, and it is thought that BRAF activation promotes ATC transformation and PIK3CA drives its growth (27). In our experience with everolimus from this case series and previously reported phase II data, PI3K/mTOR activating mutations were present in all cases with a durable response (9, 24, 25).

Interestingly, ATC rarely arises from differentiated TCs and form as metastases. In a study including 677 cases of ATC, only 6 cases demonstrated transformation at the metastatic sites (0.9%), and this cohort had the worst outcomes (28). The partial responder's ATC was first diagnosed after a lobectomy, suggesting that she had an initially poorer prognosis than the typical patient with ATC.

Our study has several limitations: although all patients who underwent treatment with everolimus off-protocol at our institution were included in this retrospective analysis, the generalizability of our findings are limited due to the sample size and sequencing data. The disease's rarity makes it challenging to study larger cohorts of patients with ATC. Some patients did not consent to genetic sequencing or did not have enough tumor tissue.

This case series adds to the existing evidence that everolimus has anti-tumor activity in ATC, and responses may correlate with mutations along the PI3K/mTOR axis. Further investigation is warranted.

## Author Contributions

EH and JL collected the data and analyzed it. All authors contributed to writing the manuscript.

### Conflict of Interest Statement

JL: Consulting, BMS; Research support, Novartis, BMS, Millennium, Bayer. The remaining authors declare that the research was conducted in the absence of any commercial or financial relationships that could be construed as a potential conflict of interest.
